# Comparison of cellular-based therapies following a long-segmental peripheral nerve defect in a rat model

**DOI:** 10.1371/journal.pone.0313292

**Published:** 2025-01-07

**Authors:** Emily L. Errante, Joseph Yunga Tigre, Ericka A. Schaeffer, Meredith C. Costello, Andrew J. Kloehn, Aiko Puerto, Aisha Khan, Yelena Pressman, Risset Silvera, Francisco J. Sanchez, Brian R. Noga, W. Dalton Dietrich, Allan D. Levi, S. Shelby Burks

**Affiliations:** 1 The Miami Project to Cure Paralysis, University of Miami Miller School of Medicine, Miami, Florida, United States of America; 2 Department of Neurological Surgery, University of Miami Miller School of Medicine, Miami, Florida, United States of America; 3 Interdisciplinary Stem Cell Institute, University of Miami Miller School of Medicine, Miami, Florida, United States of America; Julius-Maximilians-Universitat Wurzburg, GERMANY

## Abstract

Peripheral nerve injury (PNI) is characterized by a loss of cellular and axonal integrity, often leading to limited functional recovery and pain. Many PNIs are not amenable to repair with traditional techniques; however, cell therapies, particularly Schwann cells (SCs), offer the promise of neural tissue replacement and functional improvement. Exosomes, which carry cellular signaling molecules, can be secreted by SCs and have shown promise in PNI. Our laboratory has had success using SCs in preclinical and clinical treatment settings. Transplanted cells have several known limitations, which exosomes mitigate. To that end, the current study investigated if implanted SC-derived exosomes in conduits, conduits with SCs, reverse autograft, or empty conduits comparably improve axonal regeneration and pain outcomes 16-weeks after repair of a long gap PNI in adult rats. Results show that there were no differences between groups in the von Frey filament testing or in the Hargreaves test. Electrophysiological testing showed a significant difference between the injured (ipsilateral) and uninjured (contralateral) limbs while histological assessment showed a significant difference between axonal counts in different areas of the conduit. Based on the results of the current study, more research is needed to understand the therapeutic role of exosomes in PNI.

## Introduction

Peripheral nerve injury (PNI) represents 3% of trauma cases and can lead to the development of significant life-long disabilities, including neuropathy and paralysis [1, 2]. Following PNI, there is a gradual loss of supportive cells, including Schwann cells (SCs), and loss of regenerative physical and chemical guidance cues [3]. The loss of these cues occur at a faster rate than axonal regeneration, resulting in poor motor functional recovery [3]. Treatment and management are guided by the injury type and severity, with long-segmental gap defects posing significant challenges for nerve regeneration.

Autologous nerve transplantation (i.e., autografts) remain the gold standard for treatment; however, this intervention is accompanied by certain disadvantages [4]. Often, donor nerve sources, such as the sural nerve, have anatomical limitations such as lack of donor material [5]. and compromised sensory function at harvest site [6]. To address limitation of current PNI therapies, research has focused on the use of non-biological nerve guidance conduits (NGCs) to treat PNI by bridging the proximal and distal nerve ends and promote nerve regeneration via structural support [4]. In addition, there is focus on supplementing NGCs with endogenous cells critical in PNI repair [4, 7, 8], as are typically found within autografts. Following PNI, SCs transform to the repair phenotype and begin secreting various neurotrophic factors that promote cell proliferation and axon regeneration [7, 9, 10]. Our lab has previously investigated the efficacy of NGCs supplemented with SCs following large-gap PNI (13mm) and found success in functional outcome, electrophysiology, and myelination measures, comparable to the gold standard autograft [8]. However, cellular therapies, like SC implantation, elicit immunogenicity; therefore, more recent work has focused on extracting the therapeutic properties of SC, including exosomes.

Exosomes are extracellular vesicles secreted by several different cell types, including SCs, and have become an increasing area of research for PNI [11]. Exosomes contain messenger RNA (mRNA), microRNA (miRNA), and proteins that can regulate intercellular communication and recipient cellular function [9, 12, 13]. SC-derived exosomes have specifically been shown to selectively be internalized by axons and increase regenerative growth of axons, increasing peripheral nerve regeneration [14]. Importantly, compared to SCs, exosomes have low immunogenicity, allowing them to be utilized in a variety of treatment settings [15, 16]. Clinically, this is crucial, as it would allow exosomes to be used for nerve repair without a separate harvest to culture cells. Although exosome-based therapies appear to be a very promising alterative treatment, the current literature fails to evaluate long-segmental gap PNI models and long-term functional and histological outcomes. In this present study, we investigated the efficacy of NGCs supplemented with SC-derived exosomes, compared to SC-implantation and autograft, following large-gap sciatic nerve injury in rats across 16-weeks.

## Materials and methods

### Study design

Twenty-five adult male and female Fischer rats (*Rattus Fisher*; Charles River Laboratories; n = 12 males mean weight of 209 g., n = 13 females mean weight of 154 g.) were used in this study. Animals were pair housed with corncob bedding and were kept on a 12-h light/dark cycle. All procedures were approved by the Institutional Animal Care and Use Committee (IACUC) at the University of Miami. All animals had *ad libitum* access to food and water. They were housed in the veterinary facilities of our institution and were cared for according to the NIH *Guide for the Care and Use of Laboratory Animals*. Animals were randomly assigned into one of four experimental groups. The first group consisted of animals that received sciatic nerve reversed autografts (positive control), the second group received NeuraGen 3D nerve guides filled with SCs, the third group received Neuragen 3D nerve guides filled with SC-derived exosomes, and the final group received empty Neuragen 3D nerve guides (negative control). The survival time for all rats in the study was 16 weeks after surgery.

### Neuragen 3D nerve guide

The NeuraGen 3D nerve guide (Integra Lifesciences Corp.) is manufactured from a highly purified type 1 collagen derived from bovine deep flexor tendon. The collagen-glycosaminoglycan (GAG) matrix was prepared using a collagen and chondroitin-6-sulfate proteoglycan suspension [17, 18]. These nerve guides have an internal diameter of 1.5 mm and a length of 15 mm.

### SC preparation

SCs were extracted from rat sciatic nerves and purified and expanded as described previously [19, 20]. Once outward migration of fibroblasts occurred two weeks after harvest, explants were transferred onto new culture dishes, enzymatically dissociated, and then replated in DMEM/10% fetal bovine serum supplemented with 3 mitogens: bovine pituitary extract (2 mg/ml, Invitrogen), forskolin (0.8 mg/ml, Sigma), and heregulin (2.5 nM, Genentech).^7^ Cells were grown to confluency and passaged to new dishes 3 times. Their purity for grafting was found to be between 95% and 98%.

### SC-derived exosome preparation

SC-derived exosomes were derived and characterized at the Miami Project to Cure Paralysis. While rodent SCs were used, the current study elected to use human SC-derived exosomes due to reduced immunogenicity [21]. Briefly, exosomes were isolated from primary human SC cultures that were initially sourced from donor peripheral nerves (phrenic, intercostal, or lumbosacral plexus). Human SC cultures were grown using techniques as previously described [19, 20]. Following growth, cultures were washed and then underwent ultracentrifugation (centrifuged at 3 g for 10 min, filtered, centrifuged at 100,000 g for 130 min) until the supernatant containing the exosomes was aspirated.

Exosomes were confirmed through vesicle size distribution (ranging from 50-150nm) via transmission electron microscopy and a nanoparticle tracking analysis, Nanosight NS300 (Malvern Instruments Ltd.). The composition of the SC-derived exosomes was also identified using flow cytometry (CytoFLEX Flow Cytometer, Becton-Dickinson) to confirm the expression of exosomes markers CD63, CD81, and CD9. Briefly, exosomes were isolated using magnetic beads and underwent several rounds of resuspension into the isolation buffer and PBS washes. Next, the bead-bound exosomes were washed with a flow cytometry buffer (1% BSA and 0.1% sodium azide prepared in PBS) then stained for flow cytometry with fluorescent antibodies (FITC-CD63, PE-CD81 and PE-CD9) according to manufacturer recommendations. The stained cells were washed twice with the flow cytometry buffer, fixed with 4% paraformaldehyde solution, washed again with flow cytometry buffer, pelleted by centrifugation, and finally resuspended in the flow cytometry buffer to be analyzed within 24 hours of the staining.

### Operative procedure

Our surgical technique has been previously described [8, 22–25]. Briefly, all animals were anesthetized and then had their right leg shaved prior to the start of surgery. A posterior incision from the right iliac crest to the knee joint was made under aseptic conditions. The bicep femoris was reflected and the sciatic nerve beneath was dissected from the surrounding tissue. A 13 mm segment of nerve was removed via sharp transection distal to the notch and proximal to the bifurcation in all animals. This length was chosen as a severe injury model, as it is beyond the ‘critical’ gap length that has previously been noted to be greater than 10mm in the rat sciatic nerve [26]. Repair was performed according to group assignment using an operating microscope with 10–0 nylon sutures (Ethilon). The wound was closed in layers with 4–0 polyglactin sutures (Webcryl) and the skin was stapled. Staples were removed postoperative day 10. Immediately after surgery, all animals were treated with buprenorphine for pain. Apple bitter spray was used prophylactically for self-mutilation. Nylabones were added to all cages. No surgery was preformed on the left leg in any animal, allowing this leg to serve as a healthy control limb.

### SC and SC-derived exosome loading

**Fig 1:**
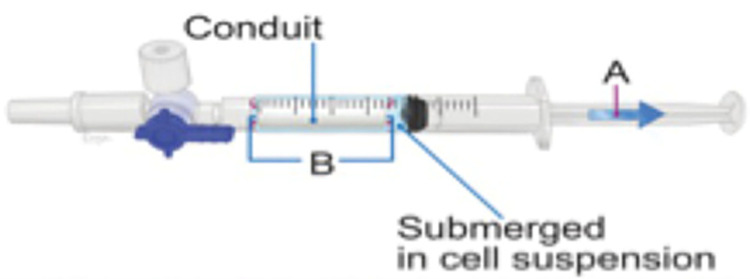
Figure showing SC and SC-derived exosome loading into NeuraGen 3D conduits. Loading into the conduit is done by first submerging the dry 15mm conduit inside of a 1-ml syringe filled with a cell solution and then applying negative pressure with a syringe plunger.

The conduit loading step occurred approximately 2–4 hours prior to surgical repair. Our loading technique has previously been described [8, 27]. Both SCs and SC-derived exosomes were first suspended in a DMEM solution at a concentration of 100,000 cells/μl and 6.66x10^11^ cells/ml, respectively. Then, a volume of 350 μl was placed into a 1-ml syringe and a dry 15 mm conduit was added to the syringe. This volume was chosen because it is the lowest possible volume that would completely submerge the conduit. Through application of negative pressure with the syringe plunger, the cells were driven into the dry conduit (Fig 1). This process was repeated for each conduit as needed.

### Autotomy scores

Beginning one day after surgery, autotomy scores were measured on a weekly basis as a measure of automutilation behavior. The scoring scale, which was previously described [28], ranges from 0 through 11. Injury of 2 or more nails was scored as 1 point, and injury of each distal half digit was scored as an additional point.

### Sensory and pain assessment

Two standard tests for nociception were used in the present study. Behavioral testing was carried out by individuals that were blind to the experimental group of each animal. All testing was done during the light cycle (6am-6pm) in a room designated for rat behavioral procedures.

#### von Frey filaments

To assess mechanical nociception, calibrated von Frey filaments ranging from 0.4 to 15 g were used [29]. Animals were put into clear Plexiglas cages on a wire mesh surface that was elevated approximately 18 inches off the table. They were allowed to habituate to the chambers for 10 minutes prior to the start of testing. Filaments were applied sequentially to both the left and the right hindpaws and kept in place for six seconds [30]. If a response was evoked, the next lowest filament was then tested; if no response occurred, then the next highest filament was tested. A response was recorded as occurring only if there was a fast withdrawal of the paw in combination with one other supraspinal pain-related behavior, such as turning their head towards the stimulus, licking, or shaking the paw. This procedure was repeated until there was a total of six completed trials. The Dixon up-down method was used to calculate a 50% paw withdrawal threshold.

#### Hargreaves test

To assess thermal nociception, Hargreaves test was used. In this test, rats were placed in clear Plexiglas cages on an elevated glass floor. An infrared emitter underneath the glass floor was placed at the center of the left or right hindpaw. When the emitter was activated, it simultaneously started a timer, which stopped when an animal withdrew its paw. These latencies were recorded. A positive response was counted when there was paw withdrawal in combination with licking the paw and moving away from the emitter. To avoid injury, in the absence of withdrawal, a cutoff time of 20 seconds was set. Animals completed three trials with 30 seconds in between each trial [31]. If the range of the responses was within approximately three seconds, all trials were kept; if it was more, additional trials were conducted.

### Electrophysiology

All animals underwent electrophysiological testing on the same day they were sacrificed. While the rat was under anesthesia, the sciatic nerve was isolated. Using a bipolar stimulator, an electrical pulse of 20 mV was applied at the sciatic notch (20 μsec, supramaximal intensity) and electromyographic signals were recorded from the gastrocnemius (GM) and tibialis anterior (TA) muscles. Peak-to-peak amplitude and onset latency were evaluated for the GM and TA for the ipsilateral and contralateral limbs.

### Muscle weight

After electrophysiology, animals were immediately sacrificed. An incision was made down to the paw. The GM was then harvested via section of the proximal and distal tendinous attachments. This was repeated on both legs. The GM from each leg was then weighed dry on a precision balance to assess percent muscle recovery.

### Histological analysis

One-micrometer plastic cross sections (n = 20) were stained with 1% toluidine blue (TB), 1% methylene blue, and 1% sodium borate solution. Sections were analyzed using an Olympus BX51 microscope at 100x magnification and Stereo investigator software (version 2019.1.2, MBF Bioscience) with an Optical Fractionator. The cross-sectional area of the epineurium was estimated by point counting with a 20x dry objective. A sampling grid of 134 μm x 134 μm with a counting frame of 30 μm x 30 μm was chosen. The total number of myelinated axons was extrapolated from the product of the area density and cross-sectional area. The randomly selected subset of counted axons was also used to determine axon diameter and myelin thickness using the Stereo investigator measure line tool (MBF Bioscience). We calculated g-ratios using the inner and outer diameters of myelinated axons. Unbiased stereological analysis was completed by three blinded investigators and averaged per animal and per group.

### Statistical analysis

Separate repeated-measure ANOVAs were preformed to assess the main effect of treatment condition, within-subjects variable (timepoint, limb, or graft location), and their corresponding interactions. Statistical assumptions, including normal distribution and homogeneity of variance/sphericity were evaluated prior to conducting tests. All data presented are represented by means and standard deviations. Orthogonal contrasts were used for post-hoc analyses. All analyses were processed through JASP 18.1 (University of Amsterdam) statistical software with an alpha cut off of 0.05. Exploratory analyses revealed no sex differences; therefore, the analysis was conducted with sex collapsed across all variables.

## Results

### Behavioral analyses

**Fig 2:**
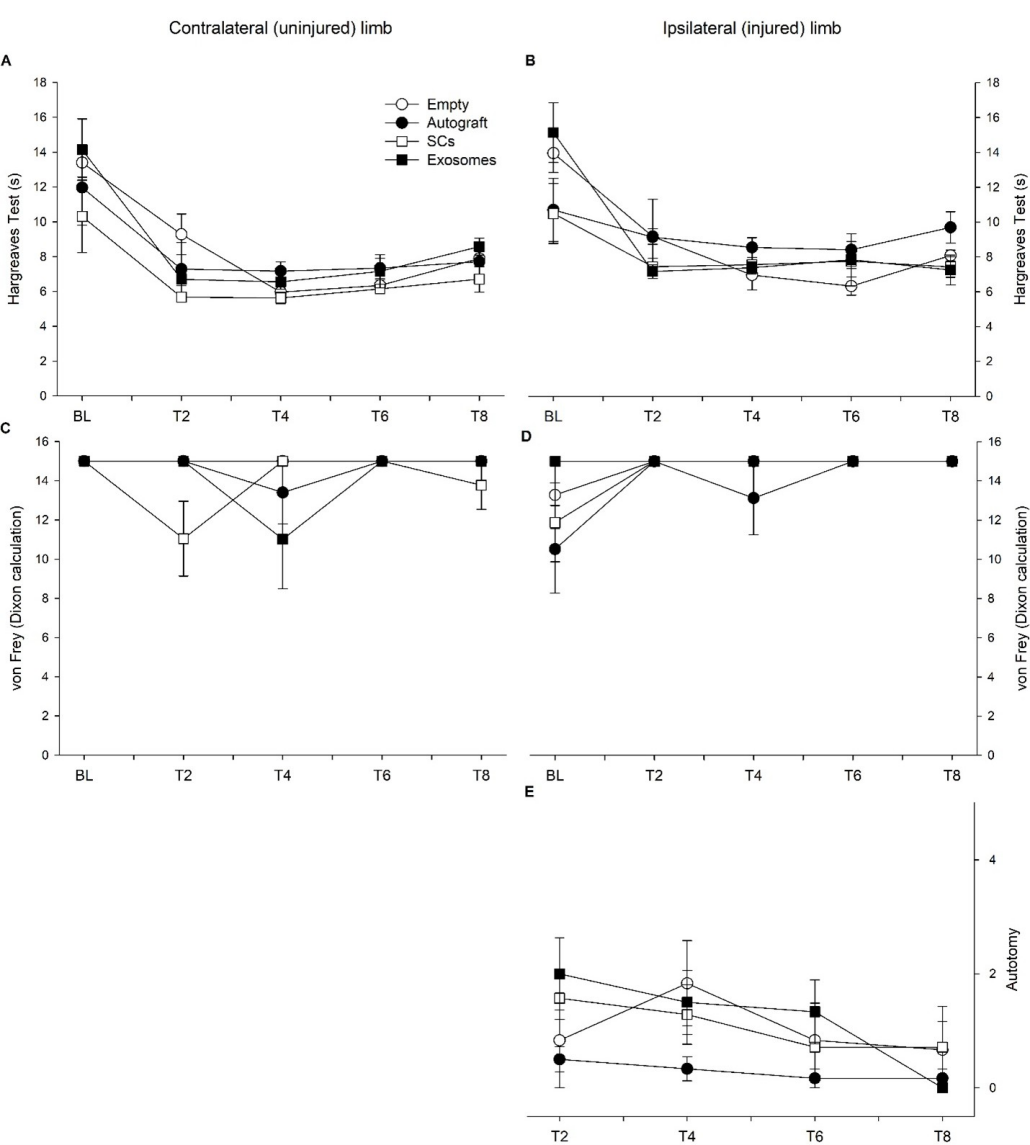
Reaction times for the Hargreaves test are plotted for the contralateral/uninjured (A) and ipsilateral/injured (B) limb across five timepoints: baseline (BL), 4-week (T2), 8-week (T4), 12-week (T6), and 16-week (T8) post-injury for each treatment condition. Additionally, von Frey scores are plotted for the contralateral (C) and ipsilateral (D) limb across five timepoints for each treatment condition. Autotomy scores are plotted for each group across four timepoints (E) and a significant effect of timepoint was observed (p = 0.041). Note: error bars represents SEM.

The Hargreaves test was used to assess heat nociception within the contralateral (uninjured) (Fig 2A) and ipsilateral (injured) hindlimb (Fig 2B). Mauchly’s test indicated a violation of sphericity (p < 0.05); therefore, a Greenhouse-Geisser correction was utilized. Repeated-measure ANOVA revealed a significant effect of timepoint (p < 0.001) and timepoint by treatment interaction (p = 0.01). Specifically, there is observed variability between the treatment groups; however, generally rats exhibited a faster reaction speed over time. Additionally, across conditions, the contralateral limb (M = 7.918 s, SD = 3.241 s) had a faster reaction than the ipsilateral, injured limb (M = 8.891 s, SD = 3.425 s; p < 0.001). Pain perception and sensitivity appears to be compromised within the injured limb.

In addition to pain perception, the von Frey filament test was used to assess mechanical nociception with higher scores indicating reduced reaction (Fig 2B and 2C). Significant effect of timepoint (p = 0.038) and timepoint by limb interaction was observed (p < 0.001) with the contralateral, uninjured, limb displaying greater fluctuation across time and the ipsilateral, injured, limb displaying reduced reaction across time. Additionally, the von Frey scores were observed to differ by treatment (p = 0.026). Orthogonal contrasts revealed no differences between the contralateral and ipsilateral limb for the empty, autograft, and exosome treatment; however, for the SC treatment, the contralateral limb (M = 13.778, SD = 3.295) was more reactive to the filaments compared to the ipsilateral limb (M = 14.654, SD = 1.928; p = 0.007).

Autotomy scores were also used to quantify sensation within the ipsilateral limb. Mauchly’s test indicated a violation of sphericity (p < 0.05); therefore, a Greenhouse-Geisser correction was utilized. A significant effect of timepoint (p = 0.041) was observed (Fig 2E). Specifically, rats initially increased autotomy scores following injury, then stabilized across time. In general, nociception appears to fluctuate across the study for each treatment condition.

### Electrophysiology and muscle analyses

**Fig 3:**
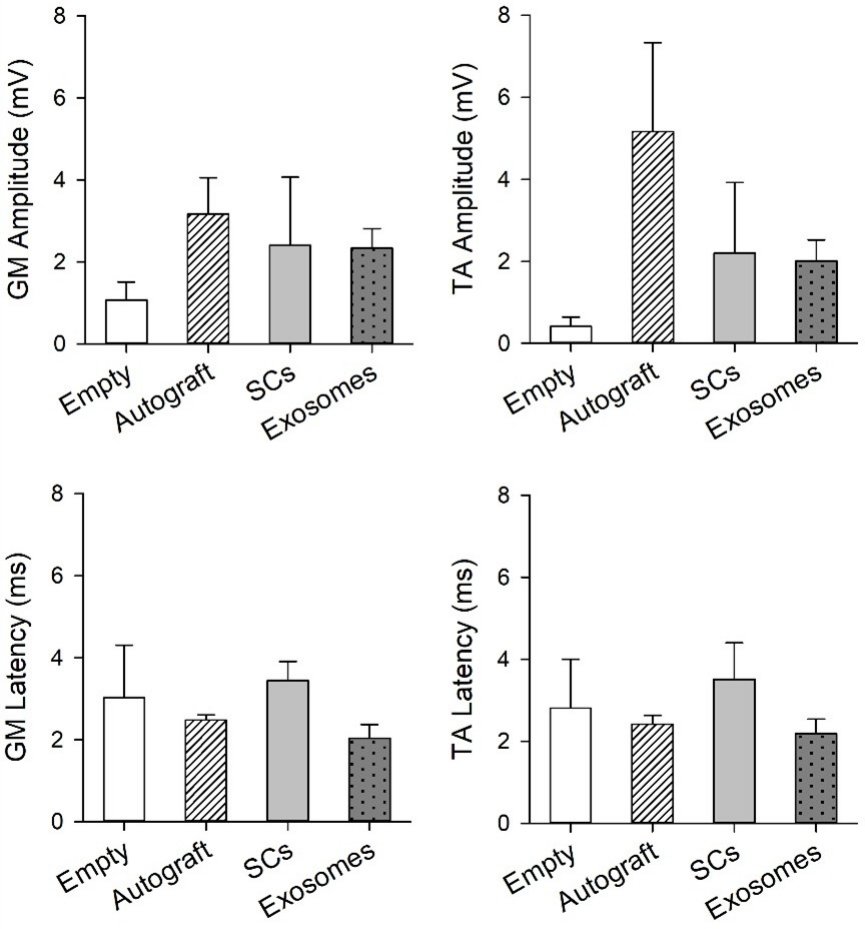
Electrophysiological characteristics of the gastrocnemius (GM) and tibialis anterior (TA) muscle from the ipsilateral (injured) limb are plotted for each treatment group. Significant differences between the contralateral and ipsilateral limb for GM amplitude were observed for the empty group (p<0.01). Note: error bars represent SEM.

The peak-to-peak amplitudes and onset latency were quantified for the GM and TA muscle in the contralateral and ipsilateral limb (Fig 3 and Table 1). Regarding amplitude, there was no observed differences for TA; however, a significant limb by treatment interaction (p = 0.026) was found for the GM. Specifically, the empty treatment had significantly higher amplitudes for the contralateral muscle (M = 4.041 mV, SD = 3.975 mV) compared to ipsilateral muscle (M = 2.0263 mV, SD = 1.834 mV; p = 0.002). Additionally, onset latency of both muscles significantly differed between limbs (GM: p = 0.003; TA: p = 0.004), regardless of treatment, with the ipsilateral limb (GM: M = 2.635 ms, SD = 1.724 ms; TA: M = 2.632 ms, SD = 1.721) displaying an increase in onset compared to the contralateral limb (GM: M = 1.376 ms, SD = 0.960 ms; TA: M = 1.318 ms, SD = 0.914). In general, electrophysiological characteristics of the injured limb vary compared to uninjured control limb.

**Table 1:** Summary of electrophysiology results.

		Empty		Autograft		SCs		Exosomes	
		Contra	Ipsi	Contra	Ipsi	Contra	Ipsi	Contra	Ipsi
Amplitude (mV)	GM	6.2 ± 3.6	1.1 ± 1.1*	1.5 ± 2.8	3.2 ± 2.0	1.9 ± 2.3	2.4 ± 3.3	3.9 ± 2.4	2.3 ± 1.4
	TA	8.2 ± 7.6	0.4 ± 0.6	10.5 ± 18.6	5.2 ± 4.9	2.2 ± 3.5	2.0 ± 3.2	2.0 ± 1.5	6.2 ± 4.2
Onset latency (ms)	GM	1.7 ± 0.9	3.0 ± 3.2	0.70 ± 1.0	2.5 ± 0.3	1.1 ± 1.3	3.4 ± 0.9	1.6 ± 0.7	2.0 ± 0.9
	TA	1.7 ± 0.8	2.8 ± 2.9	0.7 ± 1.0	2.4 ± 0.5	1.1 ± 1.3	3.5 ± 1.8	1.5 ± 0.6	2.2 ± 1.0

Values presented as mean ± SD

* Compared with contralateral limb p < 0.05

The percent difference between the contralateral and ipsilateral muscle weight was used to quantify muscle recovery. Levene’s test for equality of variances indicated a violation (p < 0.01); therefore, a Brown-Forsythe correction was utilized. Percent muscle recovery was observed to vary by treatment condition (p < 0.001) with the exosome treatment (M = 30.606%, SD = 9.858%) displaying reduced recovery compared to the empty (p = 0.003; M = 58.763%, SD = 17.179%), autograft (p < 0.001; M = 82.867%, SD = 22.0954%), and SC (p < 0.001; M = 66.305%, SD = 2.500%) groups. Additionally, the autograft treatment had significantly greater percent muscle recovery compared to the empty treatment (p = 0.009). The percent muscle recovery appeared to vary based on treatment.

### Histology

**Fig 4:**
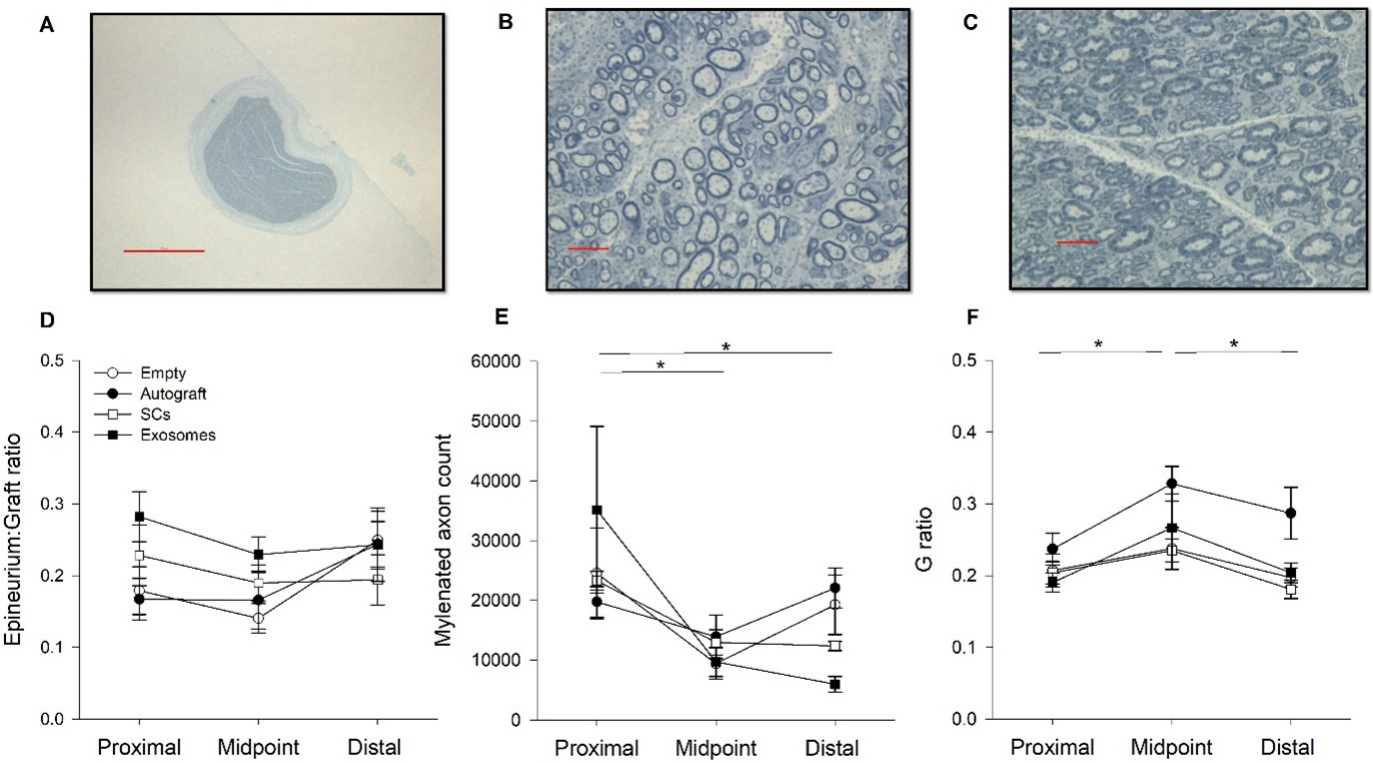
Images of the graft and myelinated axons are displayed from a representative autograft (A & C) and exosome rat (B). Results of the graft diameter to epineurium thickness (D) are plotted for each treatment condition across location of graft (proximal, midpoint, distal). The number of myelinated axons is plotted for each treatment across graft location (E) and results indicate significantly greater counts proximal, compared to midpoint or distal regions (p = 0.007). Additionally, the ratio between axon diameter and myelin thickness (g ratio) is plotted (F). The autograft group displayed thicker myelin, relative to axon diameter, compared to the other groups (p < 0.01). Furthermore, a significant effect of graft location was observed for g ratio values (p = 0.006). Note: magnification scale bar indicates 500 μm (A) and 10 μm (B & C), error bars represent SEM.

Several measures were used to quantify structural changes to the injured peripheral nerve at various locations (proximal, midpoint, distal) across treatment conditions (Fig 4A–4C). The ratio between graft diameter and epineurium thickness did not significantly vary across location or by treatment group (Fig 4D). Regarding myelin counts, Mauchly’s test indicated a violation of sphericity (p < 0.05); therefore, a Greenhouse-Geisser correction was utilized. The number of myelinated axons significantly varied across graft location (p = 0.007) with greater quantities of myelinated axons located proximally (M = 25683.31, SD = 17604.73) compared to the midpoint (p < 0.001; M = 11520.52, SD = 6005.052) and distal (p = 0.007; M = 14927.17, SD = 9043.33) regions (Fig 4E). No differences were observed between treatment conditions.

Lastly, the g ratio, ratio between axon diameter and myelin thickness, was found to significantly vary across graft location (p = 0.006) and treatment conditions (p = 0.003; Fig 4F). Specifically, thicker myelin, compared to axon diameter, was observed proximally (M = 0.21, SD = 0.043) compared to the midpoint (p = 0.003; M = 0.27, SD = 0.075). Additionally, distal g ratio values (M = 0.22, SD = 0.061) were greater than midpoint values (p = 0.009) but did not significantly differ from proximal g ratio values. Post hoc analyses revealed the autograft group (M = 0.28, SD = 0.070) had significantly higher g ratio values compared to the SC (p = 0.002; M = 0.21, SD = 0.038), exosome (p = 0.004; M = 0.22, SD = 0.070), and empty group (p < 0.001; M = 0.21, SD = 0.040). In general, myelination appears to vary by graft location and treatment condition.

## Discussion

Although PNI is common in trauma cases, treatment remains challenging, particularly when the injury is severe, such as in the case of a large gap injury. This is mainly due to a lack of autologous nerve tissue, which has led clinicians and researchers to seek improved treatment methods. NGCs have become increasingly popular as an alternative repair strategy for PNI due to their ability to provide structured support for regenerating axons and their ability to introduce cell-based therapies to the injury site. Second-generation NGCs have been shown to be particularly effective in the treatment of long-segmental injuries and have been shown to be beneficial with the use of SC therapies in PNI [8]. While SCs have been loaded into an NGC and used to treat PNI successfully, the current study was interested in assessing the ability of SC-derived exosomes delivered in a second-generation NGC to treat PNI, as measured by behavioral, electrophysiological, and histological measures. Overall, it was found that there were differences between the contralateral and ipsilateral limbs in behavioral assessments. The study also indicated that, while there were no significant differences in amplitude, there were electrophysiological differences in onset latency between the two limbs. Finally, it was also found that there were more myelinated axons in the proximal area compared to midpoint and distal and that the reversed autograft group had thicker myelin compared to all other groups.

Although the SC-derived exosome group did not significantly differ from any of the other groups, it is important to specifically note that at four months post injury, the exosome treatment group did not significantly differ from the reversed autograft group and the SC group. While other studies have examined exosomes in the context of PNI [9, 11, 12, 14], this is the first study to our knowledge that has directly compared exosomes to other treatment methods following large-gap PNI. Thus, the finding that SC-derived exosomes are comparable to SCs and reversed autograft, is critical to the development of future studies and treatment of PNI.

Interestingly, the empty conduit group, which served as a negative control, did not significantly differ from the other treatment groups across the variety of measures tested in the present study. This was unexpected when considering the lab has previously found that animals treated with SCs loaded into the conduit perform better compared to animals treated with an empty conduit [7, 8]. However, we believe that the current findings may be due to the possibility that the conduit is beneficial or the timing of the study. More specifically, the second-generation NGCs used in the current study provided structural support to regenerating axons regardless of cellular treatment used. It is possible that the empty conduit group was not significantly different than treatment groups because the axons were supported enough to regenerate across the shorter repair site, or at least to an extent such that behavioral, histological, and electrophysiological differences were not present. Additionally, it is also possible that the timeline of the study allowed for excessive regeneration through the repair site and a ceiling-effect was seen. The present study ended at 16 weeks, which is significantly longer compared to other studies in the literature [32–36]. It is possible that differences in histology or electrophysiology could have been seen at an earlier timepoint. Future studies may wish to consider a different negative control, such as a transection without repair, or use of a shorter timeline to assess differences across groups.

While we believe the current study demonstrated important findings regarding SC-derived exosomes, future studies should be conducted to further explore their potential. Specifically, different injury models should be assessed, as the present study utilized a large gap injury only. Additionally, future studies may wish to conduct a longitudinal evaluation of electrophysiology or myelin. It is of interest to understand the time course of exosome survival once introduced to the site of injury. Another variable to consider is the influence of SC and SC-derived exosomal therapy on scar tissue formation, as this is observed to be critical for the regenerative process [37]. The current study failed to evaluate scar tissue formation; however, future work may consider more specific evaluation including histological and gross anatomical measures [38]. Finally, future studies may wish to explore potential compensatory behaviors with sensory assessment of severe peripheral nerve injury, as the current study noted fluctuations in von Frey filaments across time for the uninjured limb.

In summary, a long-segmental gap injury to the rat sciatic nerve was able to be successfully repaired with second-generation NGCs filled with SCs or SC-derived exosomes, as measured by behavioral, electrophysiological, and histological analysis. Future studies should consider examining SC-derived exosomes in greater detail to better understand their role in PNI. Based on the findings from the current study, we believe exosomes have high regenerative potential in the treatment of PNI.
